# Analysis of *pmpD* Expression and PmpD Post-Translational Processing during the Life Cycle of C*hlamydia trachomatis* Serovars A, D, and L2

**DOI:** 10.1371/journal.pone.0005191

**Published:** 2009-04-15

**Authors:** Andrey O. Kiselev, Megan C. Skinner, Mary F. Lampe

**Affiliations:** 1 Department of Laboratory Medicine, University of Washington, Seattle, Washington, United States of America; 2 Division of Allergy & Infectious Diseases, Department of Medicine, University of Washington, Seattle, Washington, United States of America; Columbia University, United States of America

## Abstract

**Background:**

The polymorphic membrane protein D (PmpD) in *Chlamydia* is structurally similar to autotransporter proteins described in other bacteria and may be involved in cellular and humoral protective immunity against *Chlamydia*. The mechanism of PmpD post-translational processing and the role of its protein products in the pathogenesis of chlamydial infection have not been very well elucidated to date.

**Methodology/Principal Findings:**

Here we examined the expression and post-translational processing of the protein product of the *pmpD* gene during the life cycle of *C. trachomatis* serovars A, D, and L2. Each of these three serovars targets different human organs and tissues and encodes a different *pmpD* gene nucleotide sequence. Our quantitative real-time reverse transcription polymerase chain reaction results demonstrate that the *pmpD* gene is up-regulated at 12–24 hours after infection regardless of the *Chlamydia* serovar. This up-regulation is coincidental with the period of exponential growth and replication of reticulate bodies (RB) of *Chlamydia* and indicates a probable similarity in function of *pmpD* in serovars A, D, and L2 of *Chlamydia*. Using mass spectrometry analysis, we identified the protein products of post-translational processing of PmpD of *C. trachomatis* serovar L2 and propose a double pathway model for PmpD processing, with one cleavage site between the passenger and autotransporter domains and the other site in the middle of the passenger domain. Notably, when *Chlamydia* infected culture cells were subjected to low (28°C) temperature, PmpD post-translational processing and secretion was found to be uninhibited in the resulting persistent infection. In addition, confocal microscopy of cells infected with *Chlamydia* confirms our earlier hypothesis that PmpD is secreted outside *Chlamydia* and its secretion increases with growth of the chlamydial inclusion.

**Conclusion/Significance:**

The results of this current study involving multiple *Chlamydia* serovars support the general consensus that the *pmpD* gene is maximally expressed at mid infection and provide new information about PmpD as an autotransporter protein which is post-translationally processed and secreted outside *Chlamydia* during normal and low temperature induced persistent chlamydial infection.

## Introduction

*C. trachomatis*, an obligate intracellular bacterium, is an important human pathogen causing a variety of infections. Based on their tissue tropism, *Chlamydia* serovars are subdivided into several disease groups: ocular (serovars A-C), urogenital (serovars D-K), and Lymphogranuloma Venereum (LGV) (serovars L1-L3). Since the genome of *C. trachomatis* serovar D/UW-3 was first described in 1998, the genomes of three more *C. trachomatis* serovars, A/HAR13, L2/434/Bu, and L2b/UCH-1/proctitis, each of which targets specific human organ and tissues, have been determined [1]–[3]. Genomic analysis of these *Chlamydia* strains and *C. muridarum* (formerly *C. trachomatis* MoPn) revealed several genes including the family of the polymorphic membrane protein (*pmp*) genes with varying degrees of single nucleotide polymorphisms (SNP) which were suggested to be associated with *Chlamydia* tissue tropism and pathogenesis for each disease group [2]–[10]. In addition, recent clinical isolates and laboratory prototype strains of the same *Chlamydia* serovars that have been passaged in the laboratory for decades may also differ in their genomic composition which may influence the outcomes of *in vitro* and *in vivo* studies involving prototype strains and/or clinical isolates [11], [12]. Further, Kari *et al* [12] first demonstrated that even slight variations in genomic sequence between some ocular strains of *C. trachomatis* may have an effect on the pathogenesis of trachoma.

Alignment of the sequence of the *pmpD* gene, a member of the *pmp* family, in *C. trachomatis* serovars A, D, and L2, available in the GenBank, revealed relatively high nucleotide variability between serovar L2 and both A and D and very low variability between serovars A and D (Table 1). The protein product of this gene is structurally similar to autotransporter (AT) proteins described in other bacteria [13] and it has been shown to be significantly involved in cellular and humoral protective immunity against chlamydial infection thus, making it a prospective candidate in vaccine development [11], [14]–[18].

**Table 1:** Comparative analysis of the *pmpD* gene nucleotide sequence in *C. trachomatis* serovars A, D, and L2.

*Chlamydia* serovars whose pmpD sequence are aligned in BLAST	Total number of SNPs	Number of non-synonymos SNPs
L2/D	17*	9
L2/A	16*	10
D/A	7	3

* not including 3 nucleotide deletions in the L2 sequence

The *pmpD* gene nucleotide sequence in *C. trachomatis* serovars A, D, and L2 are available in GenBank deposited by Carlson et al [2], Stephens et al [1], and Thomson et al [3] respectively.

In our most recent publication, we demonstrated that the *pmpD* gene was up-regulated at 16–24 hours (h) post infection (p.i.) for serovar L2, which coincides with the period of exponential growth and replication of reticulate bodies (RB) [19]. This finding generally agrees with the results of other investigators who examined regulation of several chlamydial genes including *pmpD*/*pmp21* during the life cycle of *C. trachomatis* serovars D and L2 and *C. pneumoniae* CWL029 [20]–[22]. However, Nunes *et al* [11] showed that the *pmpD* gene in *C. trachomatis* serovars E and L2 was the latest (36 h p.i.) up-regulated gene among all *pmp*s, suggesting its involvement in transformation of RBs to elementary bodies (EB). Additionally, in our previous work [19], we first demonstrated that similar to bacterial AT proteins, the full length ≈160 kDa PmpD undergoes post-translational processing during the life cycle of *C. trachomatis* serovar L2 with formation of the presumable ≈120 kDa passenger domain (PD) and smaller ≈80 and 65 kDa products. We also found PmpD initially localized on the surface of RBs followed by its cleavage and secretion outside *Chlamydia*. However, the protein products of PmpD post-translational processing were not identified and the mechanism of processing was not clearly understood at that time.

While this manuscript was being considered for publication, Swanson *et al* proposed multistep processing of PmpD which results in an initial formation of *Chlamydia*-associated 73 and 82 kDa fragments and subsequent secretion of 111, 73, and 30 kDa fragments outside *Chlamydia* later in chlamydial life cycle [18]. Wehrl *et al* [16] suggested another mechanism of PmpD processing in *C. pneumoniae* which involves cleavage of PmpD with initial formation of a 130 kDa passenger domain followed by cleavage of the PD into two smaller 70 and 55 kDa fragments.

To gain further insight into the transcription of the *pmpD* gene and post-translational processing and localization of its protein product in LGV and non-LGV *C. trachomatis* strains in this current study, we examined *pmpD* transcription in serovars A, D, and L2. These three serovars were chosen because they have different human organ and tissue tropism. In addition to the prototype strains, we examined *pmpD* transcription in clinical isolates of *C. trachomatis* serovars D and L2. Using mass spectrometry (MS) analysis, we identified the protein products of post-translational processing of PmpD of *C. trachomatis* serovar L2 and proposed a mechanism of PmpD processing which is different from that described by Swanson *et al* [18]. Further, the mode of action of penicillin on PmpD post-translational processing described in our previous work [19] was not known at that time. To determine if penicillin directly suppresses serine proteases [23] or blocks the conversion from RBs to EBs [24], [25], PmpD post-translational processing was studied during persistent chlamydial infection induced by low (28°C) temperature. This condition mimics the action of penicillin by inhibiting the development of RBs [26], but has an unknown effect on proteases. Because PmpD was found to be processed at 28°C, we concluded that penicillin specifically inhibits chlamydial serine proteases including signal peptidase I involved in different stages of PmpD processing, thus supporting the general mechanism of post-translational processing of PmpD of *C. trachomatis* as that of an autotransporter protein.

## Methods

### Bacterial Strains

The *C. trachomatis* L2 prototype strain 434/Bu, L2 clinical isolate 663 passaged 13 times in our laboratory since isolation, D prototype strain UW-3/Cx, D clinical isolate 9247 passaged 18 times, and A prototype strain HAR-13 were grown in McCoy cells (ATCC CRL 1696), harvested, and purified as described previously [19]. The clinical isolates were kindly provided by R. Suchland (University of Washington, Seattle, WA). The serotype of each *Chlamydia* strain was confirmed with monoclonal antibodies as described by Suchland and Stamm [27].

### Quantification of *pmpD* gene expression by quantitative real-time RT-PCR

To study the temporal transcription of the *pmpD* gene relative to the transcription of 16S RNA in both prototype and clinical isolates of *C. trachomatis* serovars D and L2 and in the prototype strain of *C. trachomatis* serovar A, total RNA was isolated from infected McCoy cells at 2 (L2), 4, 6 (L2), 8, 12, 24, 30 (A), 36, 48, 54 (A), 60 (D), and 72 (D) hours (h) post infection (p.i.) and cDNA was synthesized as described previously [19]. Chlamydial genomic DNA was isolated from purified EBs of each strain with the DNeasy Tissue Kit (Qiagen, Valencia, CA) and used to make standards by 10-fold dilutions of known copy numbers of DNA based on its molecular mass. Real-time RT-PCR was conducted in a LightCycler Instrument (Roche Applied Science, Indianapolis, IN) using gene specific primer sets for 16S RNA [28] and *pmpD* [19]. A minimum of three PCR assays were performed for each time point using each gene specific primer set and the calculated copy numbers for each gene at each time point were averaged. Relative *pmpD* expression at each time point was calculated by dividing the average *pmpD* copy number by the average 16S RNA copy number at the same time point and multiplying by 1×10^6^ [29]. Statistical error was calculated using the standard error of the mean [29].

### Fractionation of McCoy cells infected with different *C. trachomatis* serovars

McCoy cell monolayers in six-well tissue culture plates (CORNING Inc., Corning, NY) were infected with *C. trachomatis* serovar A and D at a multiplicity of infection (MOI) of 1 and soluble and insoluble fractions were prepared at 24, 48, and 72 h p.i. and loaded onto a 10% gel as previously described [19]. In addition, a second approach was used to identify secreted proteins of *C. trachomatis* serovar L2 based on differential extraction of proteins from infected cells according to their subcellular localization by using the ProteoExtract® Subcellular Proteome Extraction Kit (EMD Biosciences Inc., San Diego, CA). Briefly, McCoy cell monolayers were infected with *C. trachomatis* serovar L2 at an MOI of 1 and at 48 h p.i., after several washes with phosphate buffered saline (PBS), cells were subsequently treated with different extraction buffers according to the manufacturer's instructions. The proteins present in each extraction buffer were precipitated with trichloroacetic acid (TCA) (Fisher Scientific, Fair Lawn, NJ), washed twice in cold acetone, and resuspended in an equal amount of Lammli buffer [30].

### 1D-PAGE and immunoblotting

After the insoluble fractions prepared at specified time points after infection were normalized against chlamydial MOMP, equal volumes of the soluble fractions were loaded onto a 10% gel as described previously [19]. The chlamydial proteins separated by SDS-PAGE were transferred to a nitrocellulose membrane, blocked, and washed according to standard procedures [31]. The membrane was incubated with pAb against the major outer membrane protein (MOMP) (Virostat, Portland, ME) or pAb against fragment 2 of PmpD (the N-terminal portion of the passenger domain of PmpD (amino acid 470–818)) [19] diluted in 2.5% non-fat milk-Trizma buffer saline-Tween 20 (NFM-TBST) and, after multiple washes in TBST, with the corresponding secondary antibody conjugated with alkaline phosphatase (AP) (Sigma-Aldrich). Blots were developed with BCIP/NBT (5-Bromo-4-Chloro3'-Indolylphosphate p-Toluidine salt/Nitro-blue Tetrazolium Chloride) according to the manufacturer's instructions (Pierce, Rockford, IL). To assess the efficiency of differential extraction of proteins from McCoy cells infected with *Chlamydia*, the proteins in each extract were probed in an immunoblot with mouse monoclonal antibodies against glyceraldehyde 3 phosphate dehydrogenase (GAPDH) (Abcam, Cambridge, MA), a host cell marker protein, followed by incubation with anti-mouse IgG conjugated with AP (Promega, Madison, WI) and development as described above.

### 2-D PAGE and immunoblotting

The proteins in the soluble fraction prepared from McCoy cells infected with *C. trachomatis* serovar L2 at 48 h p.i. were separated by 2-D electrophoresis, which was performed using the carrier ampholine method of isoelectric focusing [32] by Kendrick Labs, Inc. (Madison, WI) followed by staining the 2D gel with Coomassie Brilliant Blue R-250 (Sigma Aldrich). An identical gel, which was run in parallel with the first gel, was transblotted onto PVDF membrane followed by incubation with antibodies against fragment 2 of PmpD and development as described above. Protein spots reacting with antibodies in the immunoblot were located and excised from the Coomassie stained gel and identified by mass spectrometry fingerprinting.

### Mass spectrometry analysis and database search

MS analysis of the proteins of interest was carried out at the Protein Core Facility at Columbia University, New York, NY. Gel spots were prepared for digestion by washing twice with 100 µl 30% acetonitrile in 0.05M Tris, pH 8.5 for 20 min with shaking, then with 100% acetonitrile for 1–2 min. After removing the washes, the gel pieces were dried for 30 min in a Speed-Vac concentrator. Gels were digested by adding 0.06 µg modified trypsin (sequencing grade, Roche Molecular Biochemicals) in 13–15 µl 0.025 M Tris, pH 8.5. The tubes were placed in a heating block at 32°C and left overnight. Peptides were extracted twice with 50 µl 50% acetonitrile/2% trifluoroacetic acid (TFA) and reduced in volume in a Speed-Vac concentrator. Each dried digest was subsequently dissolved in 3 µl matrix/standard solution (10 mg/ml solution of 4-hydroxy-α-cyanocinnamic acid in 50% acetonitrile/ 0.1% TFA plus two internal standards, angiotensin I and adrenocorticotropic hormone (ACTH) 7–38 peptide) and 0.6 µl was spotted onto a sample plate. When the spot was completely dried, it was washed twice with ultra pure water. MALDI mass spectrometric analysis was performed on the digest using an Applied Biosystems Voyager DE Pro mass spectrometer in the linear mode. Instrument settings were as follows: Accelerating voltage: 20,000 V; Grid voltage: 95%; Guide wire: 0.05%; Extraction delay time: 200 nsec. Each spectrum was smoothed using Gaussian smoothing (filter width = 19 points) and calibrated using two-point calibration with the internal standards angiotensin I (1297.51) and ACTH 7-38 (3660.19). Data obtained by MALDI-TOF was searched using the ProFound protein identification program at http://prowl.rockefeller.edu/prowl-cgi/profound.exe. Average MH+ values were entered manually. Internal standards of keratin and trypsin autodigestion products at 2164.33 and 2274.60 were excluded. Search parameters were as follows: Database: NCBInr (2007/06/01); Taxonomy category: Bacteria; Protein mass range: 0–3000 kDa; Protein pI range: 0.0–14.0; Digest chemistry: trypsin, 1 max missed cuts; Modifications: +C3H5ON@C (Complete), +O@M (Partial); Tolerance: 0.50 Da

### Immunofluorecence (IMF) microscopy

To examine PmpD localization, McCoy cells grown on coverslips were infected with *C. trachomatis* serovar L2. At 16, 24, 36, and 48 h p.i., cells were fixed with 100% methanol, washed in PBST, and reacted with the mouse monoclonal antibody (mAb) LV-22 [27] against MOMP of *C. trachomatis*, rabbit pAb against fragment 2 of PmpD, or chlamydial heat-shock protein (HSP 60) (kindly provided by R. Morrison), diluted in 2.5% bovine serum albumin (BSA) (Sigma Aldrich) in PBS for 1 h at room temperature. In addition, newly generated rabbit pAb against PmpA (aa 64–548), which contrary to other Pmps lacks a signal sequence, and mouse mAb against IncA, a type III secreted protein [33] (kindly provided by D. Rockey), were also used in this study. After washing in PBS, the primary stained monolayer was reacted with goat anti-mouse IgG+IgM (H+L) antibodies conjugated with FITC (Jackson ImmunoResearch Laboratories, Inc., West Grove, PA) and goat anti-rabbit IgG antibodies conjugated with Texas Red (Molecular probes, Inc., Eugene, OR) or donkey anti-mouse IgG conjugated with Alexa 594 (Molecular probes, Inc.). Coverslips were washed, mounted, and examined using a Leica confocal system.

### Examination of PmpD post-translational processing and localization during low (28°C) temperature induced persistent *Chlamydia* infection

McCoy cells in six-well plates were infected with *C. trachomatis* serovar L2 at an MOI of 7 and, after centrifugation at 1,200×g for 1 hour, were initially incubated at 37°C for 6–8 hours to allow EBs to transform to RBs and then at 28°C for up to 192 hours after infection to inhibit RB to EB transformation [26]. To enhance cell viability during the infection, Eagle's minimal essential medium (Sigma Aldrich, St. Louis, MO) supplemented with 1 µg/ml L-glutamine, 0.5 µg/ml cycloheximide, and 10% fetal bovine serum (HyClone, Logan, UT) was replaced every 48 hours. Infected cells were rinsed with PBS then harvested at 48, 96, 144, and 192 h p.i, and soluble and insoluble fractions prepared as described previously [19]. For confocal microscopy, McCoy cells infected with *Chlamydia* and incubated at 28°C were fixed with methanol at 48 and 120 h p.i., washed in PBST, and stained with antibodies as described above. Coverslips were washed, mounted, and examined using a Leica confocal system.

## Results

### *PmpD* relative transcription in different *Chlamydia* strains

**Figure 1:**
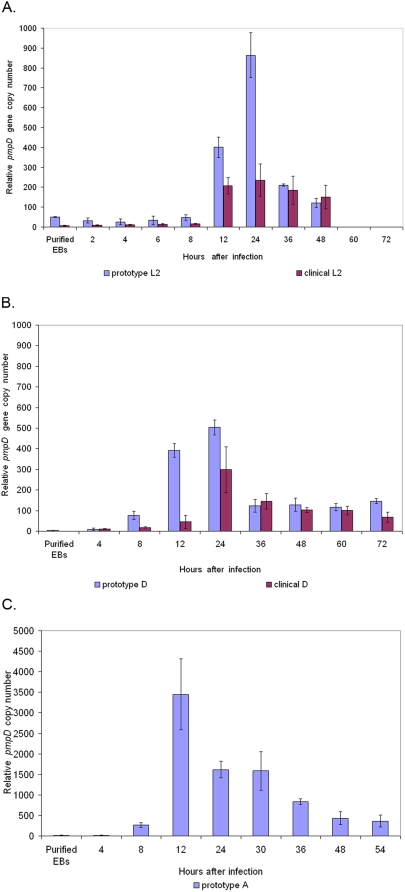
Relative *pmpD* transcription in prototype and clinical isolate strains of *C. trachomatis* serovar L2 (A), D (B), and A (C). Total RNA was isolated from *Chlamydia* infected McCoy cells at 2 (L2), 4, 6 (L2), 8, 12, 24, 30 (A), 36, 48, 54 (A), 60 (D), and 72 (D) h p.i., and from purified EBs, random primed, and assayed by real-time RT-PCR for 16S rRNA and *pmpD* copy numbers. Relative *pmpD* expression at each time point was calculated by dividing the average *pmpD* copy number by the average 16S RNA copy number at the same time point and multiplying by 1×10^6^ [29]. Error bars were based on the standard error of the mean [29]. Note that serovar A *pmpD* expression levels are much higher than levels in serovars D and L2.

The expression profile of the *pmpD* gene in prototype *C. trachomatis* serovars A, D, and L2 showed that at the early time points, up to 8 h after infection, the *pmpD* gene expression level was very low (Fig. 1) and did not significantly differ from the level observed in purified EBs. *PmpD* expression increased sharply and peaked at 12–24 h p.i. during the exponential growth and division of chlamydial RBs, then decreased but remained higher than that observed at the earliest hours after infection. In clinical isolates of serovars D and L2, *pmpD* expression followed the same general pattern as in the prototype strains, peaking at 12 (serovar L2) and 24 (serovar D) h p.i., then decreasing and/or remaining stable until the end of the course of infection. However, *pmpD* relative expression in the prototype strains during exponential growth and replication of *Chlamydia* (8–24 h p.i.) significantly exceeded that observed in clinical isolates.

### Detection of PmpD post-translational products by immunoblotting analysis

**Figure 2:**
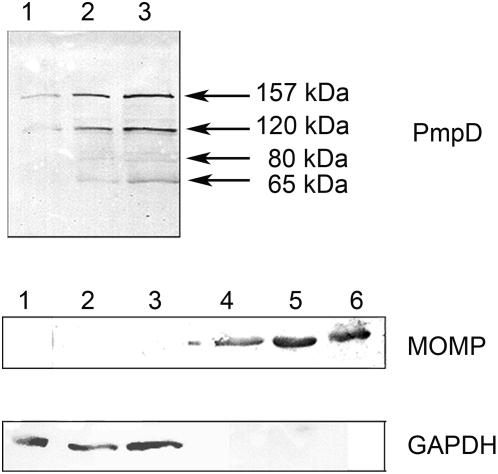
PmpD processing and secretion in *C. trachomatis* serovar A. McCoy cells infected with *C. trachomatis* serovar A were harvested at 24, 48, and 72 h p.i., and soluble and insoluble protein fractions prepared and loaded as described in METHODS. The proteins in the soluble fractions were reacted in an immunoblot with antibodies against fragment 2 of PmpD. Data not shown for PmpD in the insoluble fractions. Antibodies against MOMP and GAPDH were reacted with both the soluble and insoluble fractions to assess the efficiency of their separation. Soluble fractions: Lanes 1–3; Insoluble fractions: Lanes 4–6. Lanes 1 and 4, 24 h p.i. Lanes 2 and 5, 48 h p.i. Lanes 3 and 6, 72 h p.i.; Note that the PmpD post-translational products and the GAPDH protein are found exclusively in the soluble fractions while chlamydial MOMP is found in the insoluble fractions which contain chlamydial organisms.

**Figure 3:**
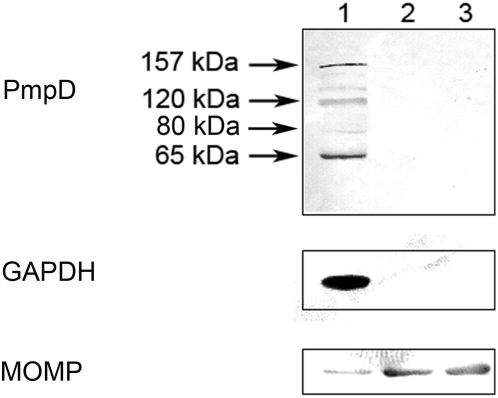
Differential extraction of proteins from culture cells based on their subcellular localization. McCoy cells infected with *C. trachomatis* serovar L2 were subsequently treated at 48 h p.i. with different Extraction buffers (Calbiochem®). The proteins in each extract were loaded in equal volumes and reacted in an immunoblot with antibodies against fragment 2 of PmpD, MOMP, and GAPDH. Lane 1, Cytosolic fraction (Extract I). Lane 2, Membrane and membrane organelles (Extract II). Lane 3, Nuclear fraction (Extract III). Note that the PmpD post-translational products and the GAPDH protein are found exclusively in the cytosolic fraction and almost all of the chlamydial MOMP is found in the subsequent fractions which contain chlamydial organisms.

The proteins in the soluble fractions prepared at specified time points after infection of McCoy cells with *C. trachomatis* serovar A were probed with antibodies against fragment 2 of PmpD in an immunoblot (Fig. 2). A ≈157 kDa protein reacted strongly at all time points examined while ≈120, 80, and 65 kDa protein bands reacted strongly only at 48 and 72 h after infection. At 24 h p.i., the 120 kDa band appeared very weak and no ≈80 and 65 kDa protein bands were detected. An identical reaction pattern was observed with proteins in the soluble fractions obtained from cells infected with *C. trachomatis* serovar D (not shown) and in previously published results with L2 [19]. No reaction was observed with a total protein lysate prepared from uninfected McCoy cells used as a negative control. As we noted in our previous work, the presence of the ≈157 and 80 kDa proteins in the soluble fractions in our experiments may be due to partial damage of the chlamydial organisms which occurred during the fractionation procedure since the cell wall of the chlamydial RBs is sensitive to mechanical factors [34]. In support of this, we found the chlamydial cytoplasmic HSP60 protein in the soluble fractions using antibodies against this protein [19]. In another approach utilizing different Extraction Buffers from a ProteoExtract® Subcellular Proteome Extraction Kit, we found the ≈120 and 65 kDa secreted PmpD post-translational products and the host cell cytoplasmic protein, GAPDH, in the material obtained at 48 h after infection with *C. trachomatis* serovar L2 which contains cytosolic proteins from the host cells and the chlamydial inclusion lumen (Extraction Buffer I) (Fig. 3). At the same time, almost all of MOMP was detected in the extracts enriched in membranes, membrane organelles, chlamydial organisms inside inclusions, and nuclear fractions (Extraction Buffers II and III respectively).

### Identification of PmpD post-translational products

**Figure 4:**
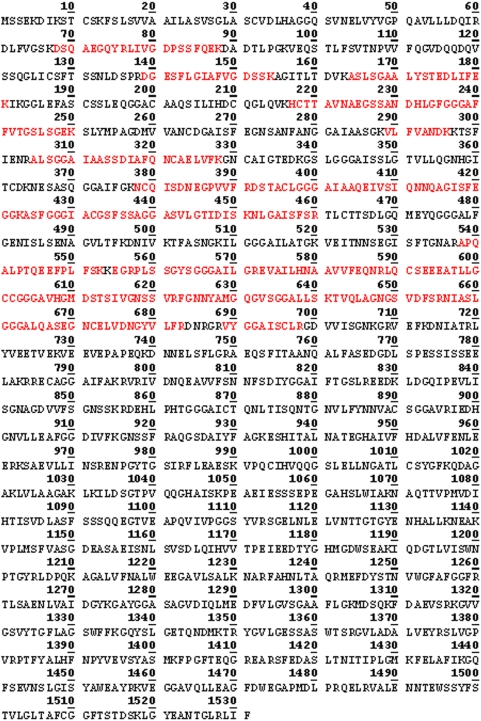
Peptides identified by MS in the ≈65 kDa protein spot (raw data). The peptides identified by MS are highlighted in red.

**Figure 5:**
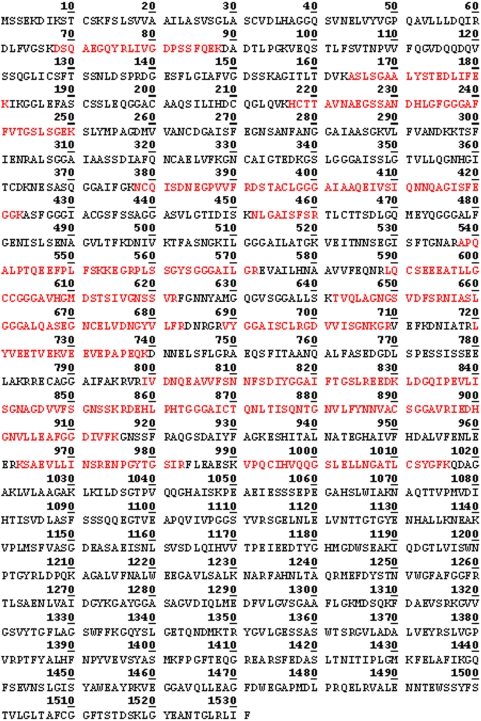
Peptides identified by MS in the ≈120 kDa protein spot (raw data). The peptides identified by MS are highlighted in red.

**Figure 6:**
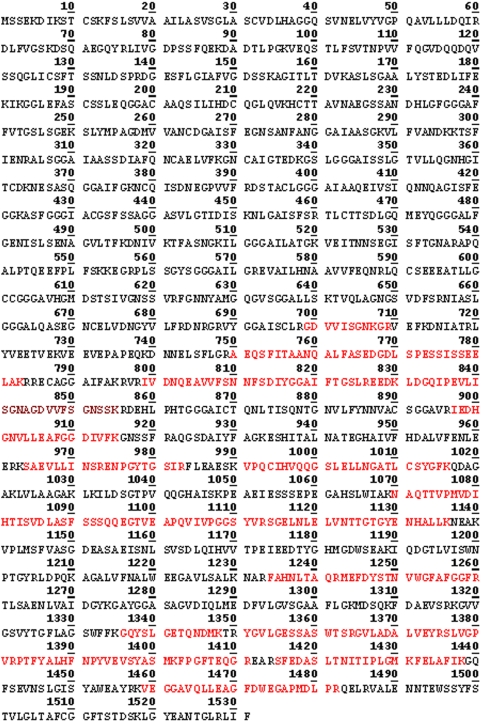
Peptides identified by MS in the ≈80 kDa protein spot (raw data). The peptides identified by MS are highlighted in red.

**Figure 7:**
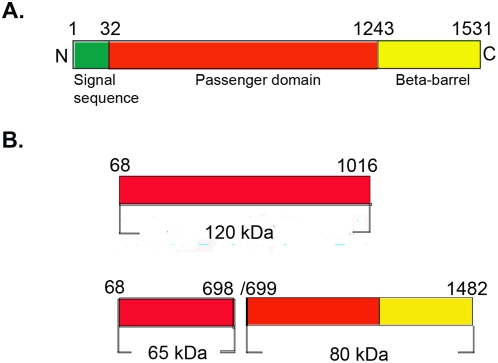
Position of PmpD post-translational products identified by MS in the PmpD aa sequence. A.The full length PmpD of *C. trachomatis* serovar L2. B. Position of the PmpD post-translational products in the PmpD sequence.

**Figure 8:**
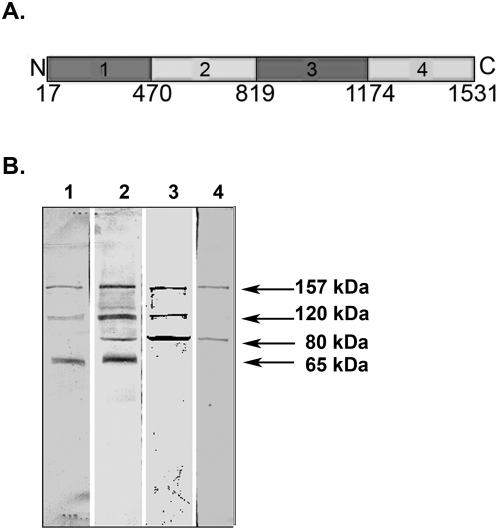
The amino acid sequence of the PmpD fragments used to raise antibodies and antibody reaction with proteins present in the soluble fraction. A. The amino acid sequence of the PmpD fragments used to raise antibodies (Fragment 1, aa 17–517; fragment 2, aa 470–818; fragment 3, aa 819–1180; fragment 4, aa 1174–1531). B. Reaction of antibodies raised against PmpD fragments with proteins present in the soluble fraction. McCoy cells infected with *C. trachomatis* serovar L2 were harvested at 48 h p.i. and the soluble fraction was prepared as described in METHODS. Lane 1, pAb against fragment 1. Lane 2, pAb against fragment 2. Lane 3, pAb against fragment 3. Lane 4, pAb against fragment 4.

In our earlier study using MS analysis, we identified one of the proteins which reacted with antibodies against fragment 2 of PmpD, the ≈157 kDa protein, as the nearly full length PmpD [19]. In this work, MS analysis of the ≈120 and 65 kDa proteins revealed that they lack the peptides located in the signal sequence and the transporter domain (TD) (the beta-barrel) (Fig 4 and 5). Moreover, the ≈65 kDa protein contained peptides identical to those located in the N-terminal half (aa 68–698) of the 120 kDa passenger domain of PmpD (aa 68–1016) (Fig 7). The peptides found in the ≈80 kDa protein began where the peptides of the ≈65 kDa protein ended and consisted of the C-terminal half of the PD and the full beta-barrel (aa 699–1482) (Fig 6 and 7). Analyzing the proteome of purified EBs of *C. trachomatis* serovars A and D, Shaw *et al* [35] found a protein with a very similar molecular weight (87.4 kDa) whose peptides “were all located in the C-terminal part” of PmpD. In addition to the results obtained from mass spectrometry, proteins present in the soluble fraction prepared after 48 h of infection of McCoy cells with *C. trachomatis* serovar L2 were probed with antibodies generated against other fragments of PmpD [19]. Each antibody reaction strongly correlated with the peptide fragment of PmpD against which the antibodies were generated (Fig. 8A). Thus, antibodies against fragment 1 did not react with the ≈80 kDa protein band, antibodies against fragment 3 did not react with the ≈65 kDa band, and antibodies against fragment 4 (the beta-barrel) reacted with neither the ≈120 nor 65 kDa proteins (Fig. 8B).

### Detection of PmpD post-translational products outside *Chlamydia* in IMF microscopy

**Figure 9:**
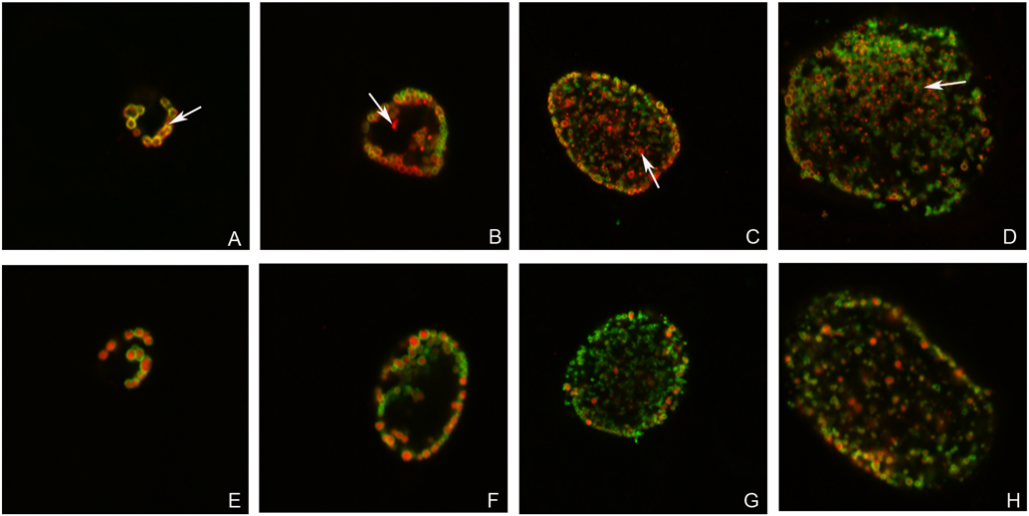
The passenger domain of PmpD is secreted outside *Chlamydia* and accumulated in the inclusion lumen during the life cycle of *C. trachomatis.* McCoy cells were infected with *C. trachomatis* serovar L2 and fixed with methanol at 16 (A and E), 24 (B and F), 36 (C and G), and 48 (D and H) h p.i. and reacted with mAb against chlamydial MOMP (green) in combination with pAb against fragment 2 of PmpD (red) (A–D) or pAb against chlamydial HSP60 protein (red) (E–H) (internal control). Arrow indicates localization of PmpD during the chlamydial life cycle on the surface of *Chlamydia* (A) or in the inclusion lumen outside *Chlamydia* (red spots) (B–D). When reacted with anti-HSP60 pAb, no material was visible outside *Chlamydia*. The photographs were made using a Leica SL confocal microscope.

**Figure 10:**
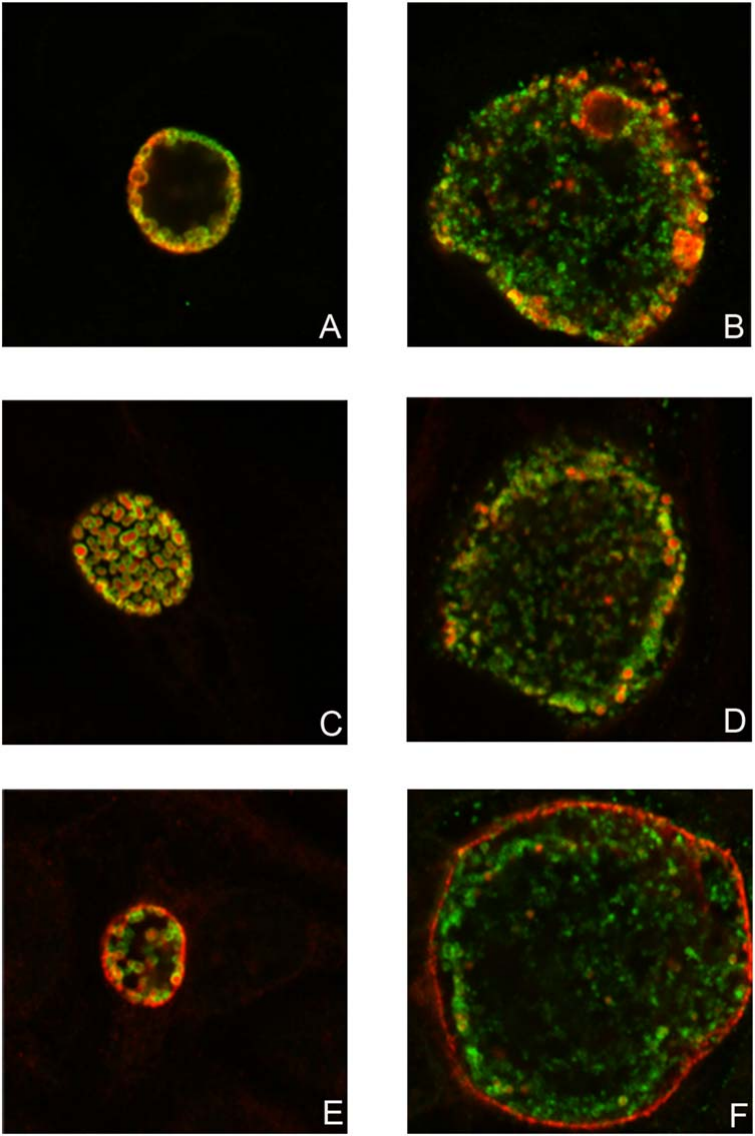
The beta-barrel of PmpD is associated with the interior of the chlamydial cell wall during the life cycle of *C. trachomatis*. McCoy cells were infected with *C. trachomatis* serovar L2, fixed with methanol at 24 (A, C, and E) and 48 (B, D, and F) h p.i., and reacted with mAb against chlamydial MOMP (green) in combination with pAb against fragment 4 of PmpD (A and B), pAb against chlamydial PmpA (C and D), or mAb against chlamydial IncA (E and F) (red). PmpA is localized in the cytoplasm while IncA is secreted outside *Chlamydia* and localized to the inclusion membrane. The beta-barrel (transporter domain) alone and included in the ≈80 kDa protein, remains associated with the interior of the cell wall of *Chlamydia* resulting in staining of chlamydial RBs with pAb against fragment 4 of PmpD in a doughnut-like pattern. Note, that contrary to pAb against fragments 1, 2, and 3, pAb against fragment 4 do not stain unfixed chlamydial RBs (not shown). The photographs were made using a Leica SL confocal microscope.

IMF microscopy of *Chlamydia* infected McCoy cells fixed at specified time points after infection and double stained with antibodies against MOMP and fragment 2 of PmpD or the HSP60 protein demonstrated that at 16 h p.i., PmpD was found strictly on the surface of chlamydial RBs (Fig. 9A). A small amount of reactive protein material was clearly visible outside chlamydial particles at 24 h p.i., but much more protein inside the inclusion lumen was visible at 48 h after infection (Fig. 9B–D), which is in agreement with the results of Swanson *et al* [18]. No such protein material was observed outside *Chlamydia* in infected culture cells stained with antibodies against MOMP (localized on the surface of *Chlamydia*), HSP60 (Fig 9E–H), PmpA (internal protein controls) (Fig 10C–D) or IncA, which is secreted outside *Chlamydia* and localized to the inclusion membrane (Fig 10E–F). Antibodies against other PmpD fragments, with the exception of fragment 4, stain McCoy cells infected with *Chlamydia* similarly to antibodies against fragment 2 (not shown). Antibodies against fragment 4 (the beta-barrel) showed that this portion of PmpD is not secreted and remains in the cell wall of *Chlamydia* (Fig 10A–B) as a part of the ≈80 kDa protein. Similarly to antibodies against fragments 1, 2, and 3, antibodies against fragment 4 stained RBs inside inclusions in host cells infected with *Chlamydia* and fixed with methanol in a doughnut-like staining pattern. However, contrary to antibodies against other PmpD fragments, antibodies against fragment 4 do not stain unfixed purified RBs (not shown), indicating that the beta-barrel is not localized on the surface of *Chlamydia*. In addition, no stained material was visible outside the inclusion.

### Examination of PmpD post-translational processing and localization during low (28°C) temperature induced chlamydial persistent infection

**Figure 11:**
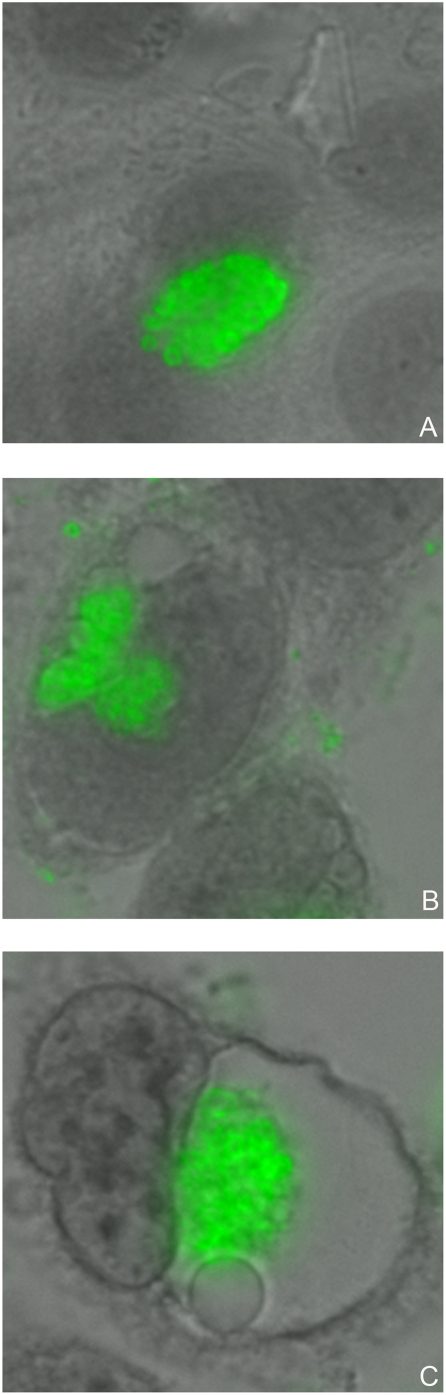
Chlamydial inclusions in culture cells produced by low (28°C) temperature induced persistent infection. McCoy cells were infected with *C. trachomatis* serovar L2, fixed with methanol at 24 (A) h p.i. at 37°C and 48 (B) and 120 (C) h p.i. at 28°C and reacted with pAb against fragment 2 of PmpD followed by staining with anti-rabbit IgG conjugated with FITC (Sigma-Aldrich) and counterstaining with Evans Blue. Multiple small non-fusogenic (B) or single “empty” looking giant (C) inclusions stained green are produced by persistent infection. A single inclusion filled with typical RBs produced by normal (37°C) infection (A) is shown for comparison. The photographs were made using Nikon Eclipse E600 upright microscope with a QImaging Retigia EX CCD Camera.

**Figure 12:**
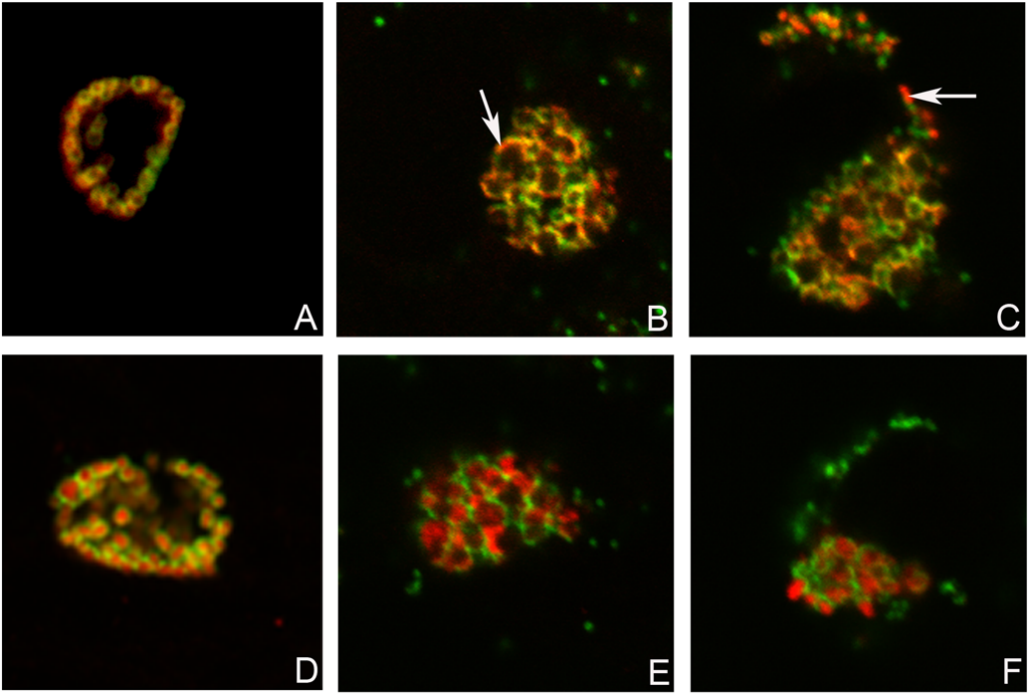
Low (28°C) temperature induced persistent infection of culture cells with *C. trachomatis* does not block PmpD post-translational processing. McCoy cells were infected with *C. trachomatis* serovar L2, fixed with methanol at 24 (A and D) h p.i. at 37°C and 48 (B and E) and 120 (C and F) h p.i. at 28°C and reacted with mAb against chlamydial MOMP (green) in combination with pAb against fragment 2 of PmpD (red) (A–C) or pAb against chlamydial HSP60 protein (red) (D–F) (internal control). RBs produced during persistent infection are abnormally large with thin cell wall (B–C and E–F) compared with uniformly sized and round shaped RBs (A and D) produced during normal infection. Arrow indicates localization of PmpD during the chlamydial life cycle on the surface of aberrant RBs (A) or in the inclusion lumen outside *Chlamydia* (red spots) (B). When reacted with anti-HSP60 pAb, no material was visible outside *Chlamydia*. Note that the rate of PmpD secretion outside *Chlamydia* is much lower and slower when compared with normal (37°C) infection. The photographs were made using a Leica SL confocal microscope.

**Figure 13:**
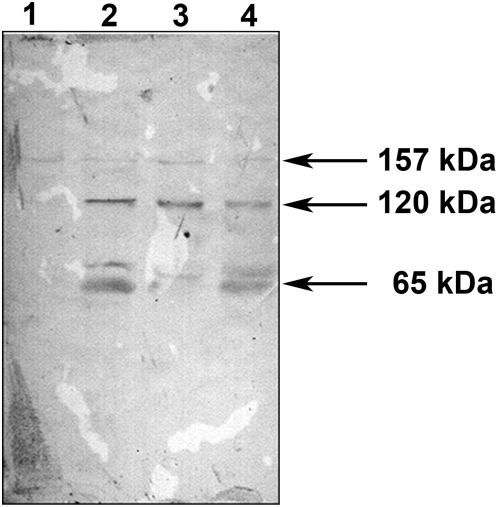
PmpD processing and secretion at 28°C. McCoy cells infected with *C. trachomatis* serovar L2 were incubated at 28°C, harvested every 48 h, and soluble fractions prepared as described in METHODS. The proteins in each soluble fraction were reacted in an immunoblot with antibodies against fragment 2 of PmpD. Lane 1, 48 h p.i. Lane 2, 96 h p.i. Lane 3, 144 h p.i. Lane 4, 192 h p.i.

*Chlamydia* infected McCoy cells were constantly monitored using inverted light microscopy during the time course of 192 h p.i., which revealed formation of multiple small non-fusogenic or single “empty” looking giant inclusions (Fig 11). IMF microscopy of infected McCoy cells fixed at 48 and 120 h p.i. and double stained with antibodies against MOMP and PmpD/HSP60 exposed inclusions filled with abnormally large aberrant RBs with a thin cell wall compared with uniformly sized and round shaped RBs produced by normal infection (Fig 12), suggesting persistent infection in culture cells. These RBs were stained in a pattern very similar to that observed in a normal infection, with HSP60 localized inside and PmpD and MOMP on the surface of RBs. In addition, a small amount of protein material stained with pAb against PmpD was clearly visible outside chlamydial RBs at 120 h p.i., indicating PmpD secretion, however at a much lower and slower rate compared with normal (37°C) chlamydial infection. An immunoblot with soluble fractions prepared from McCoy cells infected with *Chlamydia*, incubated at 28°C, and prepared at 48, 96, 144, and 192 h p.i., showed the ≈120 and 65 kDa protein bands in the 96–192 h p.i. materials reacted with anti-PmpD antibodies (Fig. 13), indicating that the PmpD protein was post-translationally processed and secreted outside *Chlamydia* and confirming the results of IMF microscopy. To ensure that the 192 h p.i. insoluble material contained no viable infectious particles (chlamydial EBs), it was used to infect McCoy cells in 96-well tissue culture plates (CORNING). Cells were stained with antibodies against MOMP and examined by IMF microscopy 48 h after infection. McCoy cells seeded with material obtained at 192 h p.i. after incubation at 28°C contained no inclusions. At the same time, chlamydial inclusions were seen in cells seeded with control material obtained from infected cells which were incubated for 48 h at 37°C.

## Discussion

To determine if the variation in the *pmpD* gene sequence in LGV and non-LGV *Chlamydia* strains leads to different *pmpD* expression patterns, we examined *pmpD* expression profiling in *C. trachomatis* serovars A, D, and L2. The *pmpD* gene transcriptional pattern with up-regulation at 12–24 h p.i. described in this study suggests that the PmpD protein may be involved in growth and division of chlamydial RBs. These results are in agreement with our earlier results [19] and those of Belland *et al* [20], Nicholson *et al* [21], and Maurer *et al* [22]. However, when Nunes *et al* examined the transcription of the *pmp* gene family in both reference and clinical strains of *C. trachomatis* serovars E and L2, they found that *pmpD* was the latest (36 h p.i) up-regulated gene, suggesting a different role for the PmpD protein in transformation from RBs to EBs and integrity of EB outer membrane [11]. We have no explanation for why our results differ from those of Nunes *et al*, but different infection conditions, dissimilar primer designs, and distinctive qRT-PCR parameters may have led to those discrepancies. Between all three serovars, *pmpD* expression was highest in serovar A and lowest in serovar D which might be explained by differential timing of the developmental phases during the life cycle of these serovars. Notably, the pattern of *pmpD* gene temporal transcription in both a clinical isolate and the prototype strain of serovar D was nearly identical, but in serovar L2 it was not. In addition, the *pmpD* relative expression in prototype strains was significantly higher than in clinical isolates during the period of exponential growth and replication of *Chlamydia*. We believe that the differences in *pmpD* expression patterns may be due to the divergence in the *pmpD* gene sequence in prototype and clinical strains or mRNA amounts which may vary from strain to strain based specific needs [11]. Because of these variations, more extensive studies involving multiple clinical isolates are needed in order to draw a definite conclusion about any trends in the *pmpD* gene transcription pattern in clinical isolates versus prototype strains.

When the PmpD protein post-translational processing pattern was examined during the developmental cycle of *C. trachomatis* serovars A and D, it was found to be very similar to that observed in serovar L2 [19]. The results of MS analysis indicated that the creation of the ≈120 kDa protein represents classical autotransporter pathway of processing of PmpD with a cleavage site between the PD and TD (aa 1242 and 1243) recognized by outer membrane proteases. However, the peptide composition of the ≈65 and 80 kDa proteins led us to the speculation that an additional/alternative cleavage site located in the middle of the PD (between aa 698 and 699) is recognized in order to allow its secretion outside bacteria [36]–[39]. There is a possibility that the ≈65 and 80 kDa proteins resulted from cleavage of PmpD by another AT protein with serine protease-like activity [40] since the peptide bond between aa 698 (arginine) and 699 (glycine) represents one of the cleavage sites recognized by serine proteases [41]. The IMF microscopy with pAb against different PmpD fragments demonstrated that the PD is linked to the surface of RBs prior to its secretion into the inclusion lumen and the TD which is a part of the ≈80 kDa protein, remains inside the cell wall of *Chlamydia*. Interestingly, the ≈30 kDa protein band correlating to the cleaved TD of PmpD was never found in our experiments suggesting its quick degradation due to potential high toxicity of the beta-barrel to bacteria [42], [43].

The PmpD post-translational products identified in this work (≈120, 65, and 80 kDa fragments) are very similar to those described by Swanson *et al* (111, 73, and 82 kDa fragments) [18]. However, Swanson *et al* [18] proposed the cleavage of PmpD with the primary formation of *Chlamydia*-associated 73 and 82 kDa fragments starting at mid (24 h p.i.) infection and secondary formation of a soluble 111 kDa fragment followed by its cleavage and subsequent production of 73 and 30 kDa proteins occurring at late (30–48 h p.i.) infection. Contrary to their findings, we found the PD of PmpD (the ≈120 kDa protein) secreted as early as 24 h p.i. Two additional PmpD post-translational products which resulted from non-conventional processing, the ≈65 kDa and 80 kDa fragments, appear at later time points after infection (30–48 h p.i.). Notably, the soluble 30 kDa segment of PmpD first described by Swanson *et al* [18], never reacted in an immunoblot with antibodies employed in our studies which could be due to recognition of different epitopes in *Chlamydia* by antibodies generated in our study. The mode of PmpD processing suggested in our work is supported by that of Wehrl *et al* [16], who also proposed the earlier cleavage of the PD of PmpD during the life cycle of *C. pneumoniae*. Notably, confocal microscopy demonstrated staining of the protein material outside of chlamydial organisms in the inclusion lumen with the anti-PmpD pAb starting at 24 h after infection, thus confirming the timing of PmpD secretion in an immunoblot.

To investigate the mode of action of penicillin in our previous work [19], PmpD post-translational processing was examined at low (28°C) temperature. To our knowledge, this is the first study describing expression of a Pmp during persistent infection. We demonstrated that the incomplete chlamydial developmental cycle which occurs during low temperature induced persistent infection does not prevent PmpD post-translational processing. Thus, it appears that penicillin specifically inhibits membrane proteases including signal peptidase I and other serine proteases which cleave an autotransporter protein during its multistep processing [23], [44] resulting in blockage of PmpD processing [19]. Associated with altered development during *Chlamydia* life cycle, persistent infection is considered a significant aspect in the pathogenesis of chlamydial infection [45], [46]. Our findings that the PmpD processing and secretion outside *Chlamydia* are not inhibited during low temperature induced persistent infection indicate that PmpD and its post-translational products may play an important role in survival of *Chlamydia* in harsh environmental conditions contributing to mediation between the host cell and *Chlamydia*. However, the PmpD expression pattern during persistent infection could be different depending on the factors inducing this infection. For instance, Klos *et al* [47] and Goellner *et al* [48] demonstrated that persistent infection induced by variety of factors resulted in dissimilar transcription pattern of a range of genes in *C. pneumoniae* and *C. psittaci* respectively. In addition, it was shown that another important component of the chlamydial outer membrane, the MOMP protein, was so greatly reduced during IFN-γ induced infection that anti-MOMP mAb did not stain chlamydial inclusions in culture cells [49], though in our study, *Chlamydia* organisms were clearly stained with anti-MOMP antibodies. More experiments need to be carried out to explain the role of chlamydial Pmps in the pathogenesis of persistent chlamydial infection.

In summary, the variability in the nucleotide sequence of the *pmpD* gene of C. trachomatis serovars A, D, and L2, which have different tissue tropisms, did not lead to significant dissimilarities in the *pmpD* gene transcription profiling and the PmpD protein post-translational processing pattern, thus indicating that the *pmpD* gene may have similar functions in all three *Chlamydia* serovars. In addition, the peptide profile of the PmpD post-translational products, the mechanism of their formation, and their localization during the chlamydial life cycle are in agreement with the general theory that PmpD is an autotransporter protein.
